# A Rare Case of a Fenestrated Retromandibular Vein Lying Lateral to the Facial Nerve Bifurcation

**DOI:** 10.7759/cureus.50973

**Published:** 2023-12-22

**Authors:** Alexandros Poutoglidis, Paraskevi Karamitsou, Stefanos Triaridis, George K Paraskevas, Georgios Langas, Stavros Tsiakaras, Ioannis Mykoniatis, Elpis Chochliourou, Nikolaos Lazaridis

**Affiliations:** 1 Department of Anatomy and Surgical Anatomy, School of Medicine, Aristotle University of Thessaloniki, Thessaloniki, GRC; 2 Department of Otorhinolaryngology/Head and Neck Surgery, 'G. Papanikolaou' General Hospital, Thessaloniki, GRC; 3 Department of Otolaryngology/Head and Neck Surgery, School of Medicine, Aristotle University of Thessaloniki, AHEPA University Hospital, Thessaloniki, GRC; 4 1st Department of Urology, School of Medicine, Aristotle University of Thessaloniki, 'G. Gennimatas' General Hospital, Thessaloniki, GRC

**Keywords:** anatomy, cadaver, head and neck, retromandibular vein, facial nerve

## Abstract

Facial nerve integrity is the cornerstone of parotid surgery. Although a variety of anatomical landmarks have been employed, facial nerve injury still happens causing devastating functional and cosmetic sequelae. The retromandibular vein is considered one of the most consistent structures lying just deep into the facial nerve. In our cadaveric study, we found a fenestrated retromandibular vein lying superficial to the bifurcation of the facial nerve. This rare anatomical variation would have been a challenge for a hypothetic parotidectomy. Surgeons should be aware of both anterograde and retrograde dissection of the facial nerve and choose the most proper approach to preserve the integrity of the facial nerve.

## Introduction

The preservation of the integrity of the facial nerve during parotid surgery is of paramount importance. Permanent facial nerve palsy is considered a devastating complication having both cosmetic and functional implications [1]. Nowadays, parotid surgery is performed with the assistance of a facial nerve stimulator to avoid an inadvertent injury. In addition, surgical landmarks such as tragal pointer, tympanomastoid suture, posterior belly of the digastric, and many others are used to help with the identification of the facial nerve and secure a safe dissection plan [2].

The extratemporal segment of the facial nerve has a complex branching pattern with multiple anastomoses among its terminal branches. In 1956, Davis et al. conducted a large cadaveric study and proposed six distinct branching patterns [3]. Although new proposals arose through the years, Davis's classification was proven to be accurate and simple through a systematic review of a thousand cases [4]. The relationship of the facial nerve with adjacent tissues is also unstable making parotid surgery more challenging [5]. The retromandibular vein crosses the parotid gland just deep to the facial nerve and consists of the radiological landmark between the superficial and the deep lobe of the parotid gland.

Our case study aims to present a rare anatomical variation of a fenestrated retromandibular vein lying lateral to the facial nerve bifurcation. The study presents the variation of the relationship between the retromandibular vein and the facial nerve that is not associated with the facial nerve branching pattern.

## Case presentation

**Figure 1:**
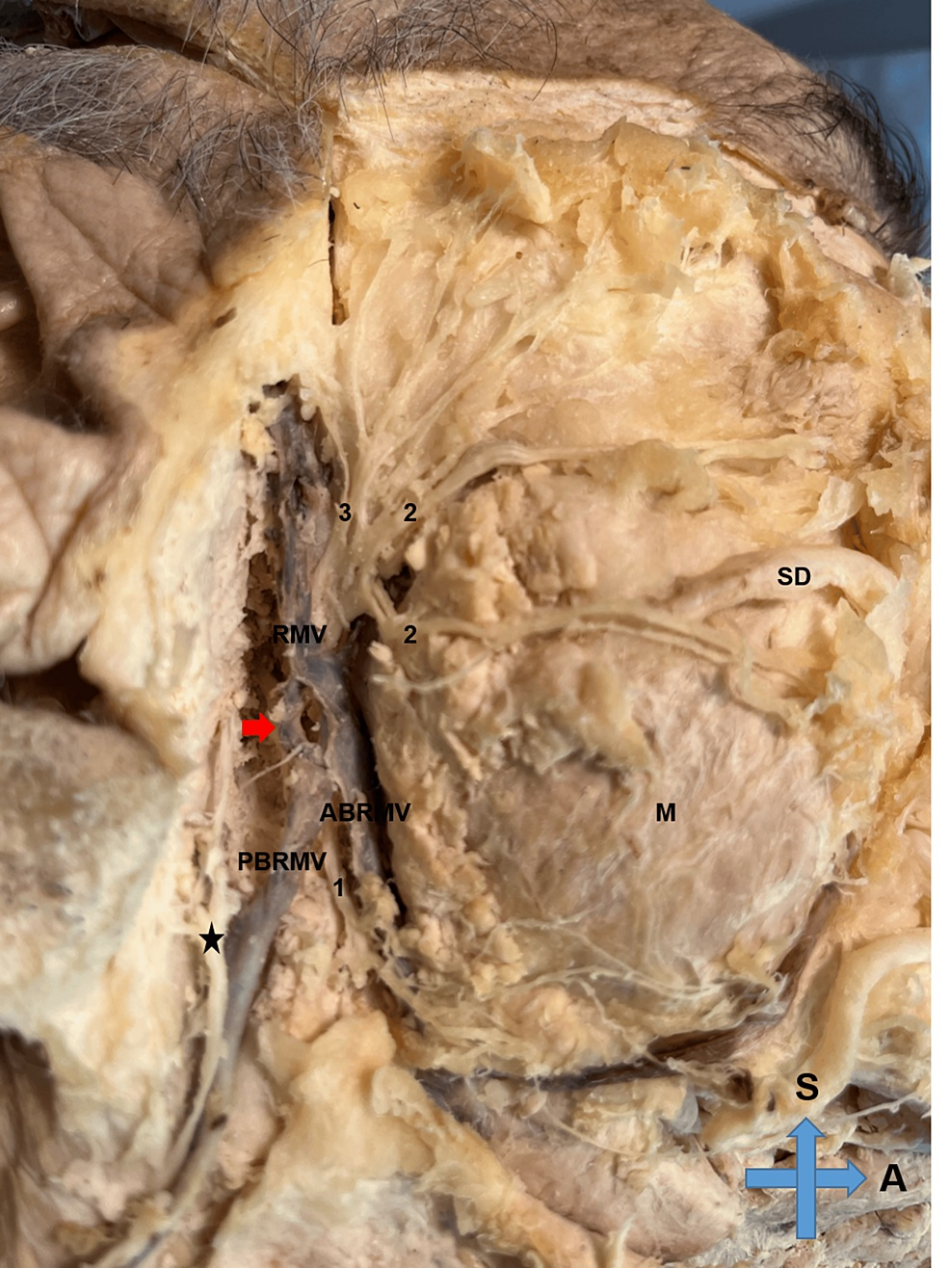
Anatomical variation of the retromandibular vein with fenestration in the middle part RMV: retromandibular vein, ABRMV: anterior branch of the retromandibular vein, PBRMV: posterior branch of the retromandibular vein, M: masseter muscle, SD: Stensen duct, S: superior, A: anterior, *: greater auricular nerve, 1: mandibular branch of the facial nerve, 2: buccal branches of the facial nerve, 3: temporofacial branch of the facial nerve, red arrow: fenestration of the retromandibular vein

A very rare anatomical variation of the retromandibular vein and its relationship with the facial nerve was observed during the dissection of a 78-year-old Caucasian formalin-embalmed male cadaver from Northern Greece. The cadaver was donated to the Department of Anatomy and Surgical Anatomy of Aristotle University of Thessaloniki in Greece for educational purposes. Cardiopulmonary arrest was recorded as the cause of death. Dissection of the cadaver revealed a skin growth in the contralateral hemiface and no gross pathology on the ipsilateral side. The facial nerve was identified distally and dissected retrograde through its mandibular branch. The retromandibular vein was lateral to the bifurcation of the facial nerve and not medial as it was expected. There was also a fenestration in the middle part of the vein with no neurovascular structures crossing it (Figure 1).

**Figure 2:**
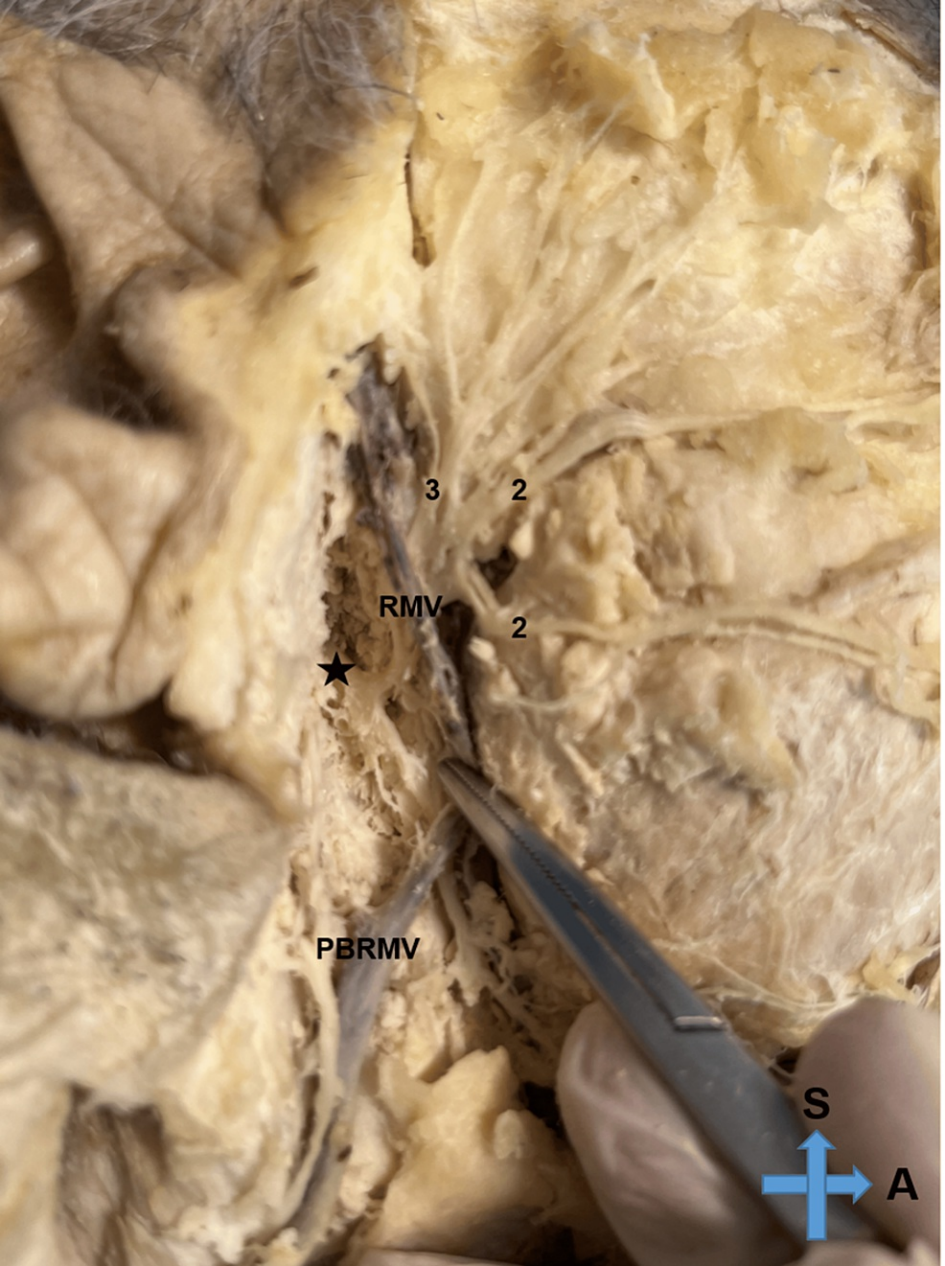
Retraction of the retromandibular vein revealing the main trunk of the facial nerve with its bifurcation RMV: retromandibular vein, PBRMV: posterior branch of the retromandibular vein, S: superior, A: anterior, *: greater auricular nerve, 2: buccal branches of the facial nerve, 3: temporofacial branch of the facial nerve

Gentle retraction of the vein revealed the main trunk of the facial nerve with its bifurcation (Figure 2).

The cervicofacial division of the facial nerve crossed over the anterior division of the retromandibular vein at the point of the angle of the mandible 3.52 cm after the bifurcation point. The fenestration of the retromandibular vein was 2.61 cm inferiorly to its upper part and 1.24 cm superiorly to its bifurcation. The largest diameter of the fenestration was 0.22 cm. The measurements were conducted by two independent observers and the median values were employed for the analysis. The interclass correlation coefficient (ICC) was used to ensure intraobserver and interobserver reliability.

## Discussion

Parotid gland surgery requires excellent knowledge of the surgical anatomy of the facial nerve to avoid iatrogenic facial nerve palsy. However, surgical landmarks and nerve stimulators help surgeons to orientate during surgery; the unpredictability and the anatomical variations of the facial nerve put the integrity of the nerve at risk.

The relationship of the facial nerve with the retromandibular vein has been documented and some authors proposed classification systems. In 1995, Kopuz et al. presented a cadaveric study of 45 hemi-faces and found that in most cases retromandibular vein was lateral to the facial nerve and in some cases was medial to the lower division of the nerve. He proposed a classification of six patterns. One of these patterns included a retromandibular vein lateral to the facial nerve bifurcation [6].

In 2022, Khan et al. performed a surgical study of 70 parotid surgeries. They identified four abnormal relationships between the facial nerve and the retromandibular vein. However, in all these cases, the facial nerve was never totally lateral to the retromandibular vein [7].

Fenestrations of head and neck veins are rare. According to some authors, fenestration of the internal jugular vein is present in 0.4% of the population as an anatomical variation [8]. External jugular vein fenestration has been reported rarely [9,10]. To the best of our knowledge, fenestration of the retromandibular vein has not been reported yet. Paraskevas et al. presented a scarce case of a fenestrated external jugular vein where the cervical cutaneous vein was crossing through the fenestration [10].

The retrograde dissection in parotid surgery is used for tumors superficial to the main trunk of the nerve. It is an uncommon alternative to the classical anterograde approach and is usually performed after the identification of either a buccal or a mandibular branch of the facial nerve [11]. According to a study, only a few surgeons can perform this [12].

Our case would have been a nightmare scenario for a parotid surgeon. The bifurcation of the facial nerve medial to the retromandibular vein makes its identification extremely difficult. Ligation of the retromandibular vein would have been unavoidable to dissect the nerve trunk with an anterograde approach. However, an experienced surgeon could have changed his approach by performing the dissection retrogradely.

## Conclusions

Parotid gland surgery requires training and experience. Excellent knowledge of the facial nerve anatomy and its variations is imperative to perform a parotidectomy. However, retromandibular vein is considered one of the most consistent landmarks; it may present with variations that increase the risk of intraoperative complications. The surgeon should be able to perform the surgery using both anterograde and retrograde approaches to minimize the risk of a facial nerve injury.
